# Drosophila RecQ4 Is Directly Involved in Both DNA Replication and the Response to UV Damage in S2 Cells

**DOI:** 10.1371/journal.pone.0049505

**Published:** 2012-11-16

**Authors:** Gilles Crevel, Nicole Vo, Isabelle Crevel, Sana Hamid, Lily Hoa, Seiji Miyata, Sue Cotterill

**Affiliations:** 1 Department Basic Medical Sciences, St. Georges University London, Cranmer Terrace, London, United Kingdom; 2 Department of Applied Biology, Kyoto Institute of Technology, Kyoto, Japan; University of Medicine and Dentistry of New Jersey, United States of America

## Abstract

The RecQ4 protein shows homology to both the *S.cerevisiae* DNA replication protein Sld2 and the DNA repair related RecQ helicases. Experimental data also suggest replication and repair functions for RecQ4, but the precise details of its involvement remain to be clarified.

Here we show that depletion of DmRecQ4 by dsRNA interference in S2 cells causes defects consistent with a replication function for the protein. The cells show reduced proliferation associated with an S phase block, reduced BrdU incorporation, and an increase in cells with a subG1 DNA content. At the molecular level we observe reduced chromatin association of DNA polymerase-alpha and PCNA. We also observe increased chromatin association of phosphorylated H2AvD - consistent with the presence of DNA damage and increased apoptosis.

Analysis of DmRecQ4 repair function suggests a direct role in NER, as the protein shows rapid but transient nuclear localisation after UV treatment. Re-localisation is not observed after etoposide or H_2_O_2_ treatment, indicating that the involvement of DmRecQ4 in repair is likely to be pathway specific.

Deletion analysis of DmRecQ4 suggests that the SLD2 domain was essential, but not sufficient, for replication function. In addition a DmRecQ4 N-terminal deletion could efficiently re-localise on UV treatment, suggesting that the determinants for this response are contained in the C terminus of the protein. Finally several deletions show differential rescue of dsRNA generated replication and proliferation phenotypes. These will be useful for a molecular analysis of the specific role of DmRecQ4 in different cellular pathways.

## Introduction

Rothmund –Thomson syndrome, Baller-Gerold syndrome and RAPADILINO syndrome are three recessive genetic disorders which are characterised by a disparate array of symptoms including skin degeneration, growth deficiency, skeletal abnormalities and high predisposition to osteosarcomas. Although the precise mechanism by which these symptoms are generated is unclear, one protein which has been seen to be mutated in a high percentage of cases is the RecQ4 protein [1], [2].

RecQ4 is classified as part of the RecQ family of helicases [3]. In addition the N terminal region shows weak homology to the yeast SLD2 protein [4] - a central protein in the control of the initiation of DNA replication. This has led to the suggestion that this protein has dual functions in DNA replication and repair, and recent studies have provided experimental evidence to support this.

In support of a replication role for RecQ4, Xenopus extracts which are lacking RecQ4 show decreased BrdU incorporation [4]–[5], and depleted mammalian cells show proliferation defects [4]. Further evidence is provided by the physical and functional interaction of RecQ4 with replication proteins. In Xenopus extracts RecQ4 appears to directly interact with Cut5 but not Mcm2-7 or Cdc45 [4]–[5]. It loads onto chromatin at the same stage of the cell cycle as Cut5, and its loading requires preRC formation. In addition depletion of RecQ4 causes a decrease in the loading of RPA and DNA polymerase alpha onto chromatin, but has no effect on Mcm2-7, Cdc45, Cut5, pol epsilon, or GINS loading. Mammalian RecQ4 does not apparently interact with Cut5, but does show interactions with Mcm2-7, Mcm10, Cdc45, and GINS [6]–[7]. Loss of RecQ4 causes decreased binding of GINS, although the binding of Mcm7, Mcm10 and CDC6 are not affected. It has also been reported to load at the lamin b origin [6]. Mouse knockouts which interfere with the RecQ like helicase domain are viable [8], but a disruption near the SLD2 homology domain is lethal [9]. These data clearly suggest a replication role for RecQ4, but inconsistencies in the reported protein interactions complicates interpretation of the precise replication function of RecQ4.

In support of a repair role for RecQ4, genomic instability is observed in both patient cells and mouse models [10]. In addition Hydroxyurea (HU), camptothecin (CPT), doxyrubicin (DOX), cis-platin (CDDP) UV, ionizing radiation (IR) and hydrogen peroxide (H_2_O_2_ sensitivity of patient cells has been reported in some studies eg [11]–[12] [2] (although discrepancies with sensitivity are observed between different studies/cell lines eg [13]). More specific studies from different labs have suggested that RecQ4 may function in three different repair pathways: A role in NER is suggested by the observation that after UV damage the protein is seen to bind to chromatin foci and interact with XPA [14]. If RecQ4 is not present the damage is reported to remain unrepaired: Etoposide treatment also causes increased focal chromatin binding and an interaction with Rad51, suggesting a role in dsb repair [15]: Finally BER induced by H_2_O_2_ treatment causes co localization with APE1 and FEN1 [16], and in vitro RecQ4 stimulates APE1 nuclease activity. The exact mechanism by which RecQ4 functions in any of these repair pathways remains to be determined.

Unlike most other eukaryotes which have five RecQ4 helicases Drosophila has only three; BLM, RecQ4 and RecQ5. It is therefore possible that DmRecQ4 may have additional functions compensating for the lack of WRN and RecQ1. In fact a comparison of protein sequences suggests that DmRecQ4 has a 382aa region (aa228-610) that is not present in RecQ4 proteins from other species. Previous studies in whole flies have again suggested replication and repair involvement for DmRecQ4. A replication role is supported by the observation that in targeted gene knockouts larval brains show decreased proliferation and BrdU incorporation, with cells apparently stopping at the G2/M boundary [17]. In addition hypomorphic mutants generated by imprecise p element excision showed decreased amplification of the chorion locus in follicle cells during oogenesis [18]. Repair function is suggested by an apparent increased sensitivity to paraquat, gamma radiation and a single induced chromosomal DSB in mutant flies [17]. Although these studies have suggested involvement in replication and repair, the use of whole flies complicates the analysis of precise biochemical interactions due to the variety of cell types present and interactions of replication and repair with developmental pathways.

Here we present our analysis of Drosophila S2 cells as a model for the detailed study of DmRecQ4. We confirm a replication role for the protein, which requires the presence of the SLD2 domain of the protein, and analyse its interactions with other replication proteins. We also look at its involvement in NER, DSB repair and BER. We find evidence for a direct role in the response to UV damage, and suggest that any role in DSB repair and BER must take place via a significantly different mechanism.

## Results

### DmRecQ4 Depleted S2 Cells Show Decreased Proliferation Due to an S Phase Block

**Figure 1:**
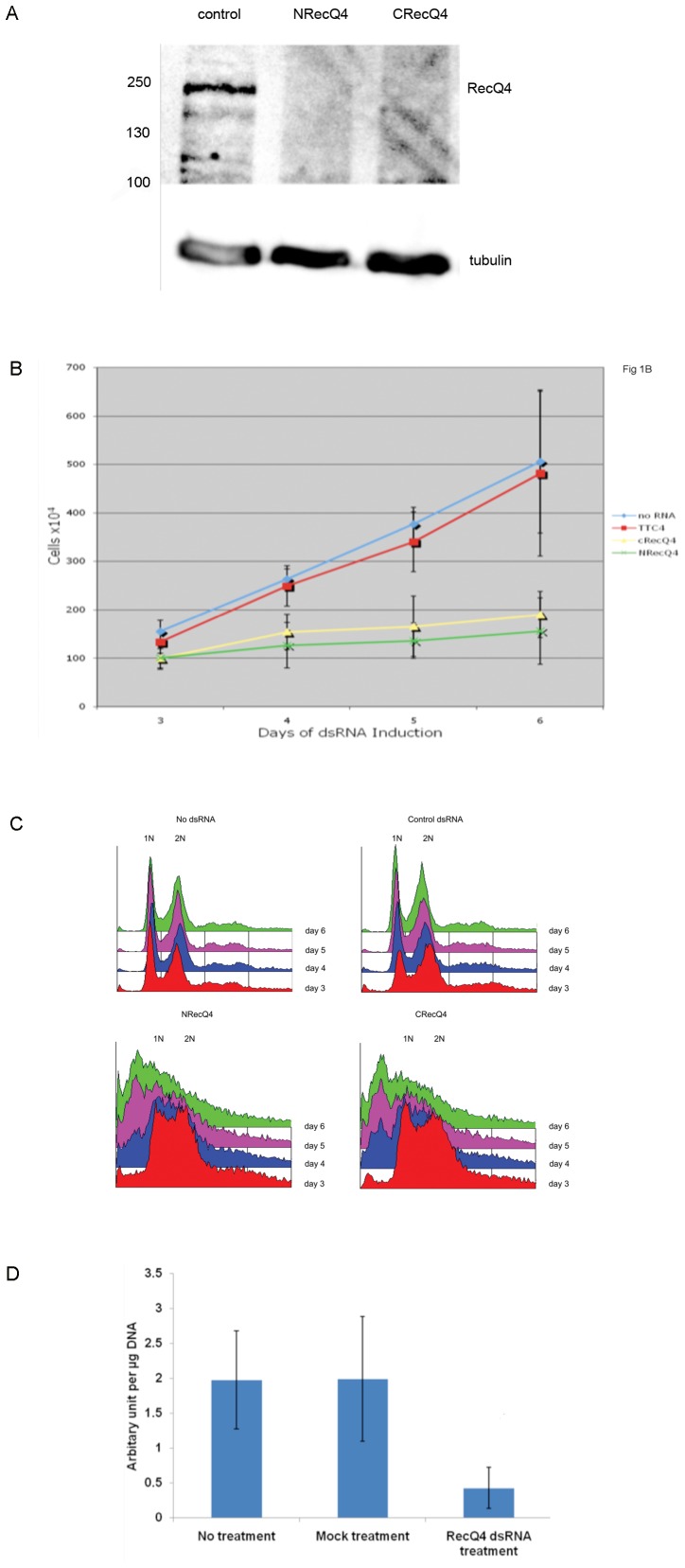
Reduction of DmRecQ4 in S2 cells interferes with cell proliferation and DNA synthesis. A) Total extracts of S2 cells which had been mock depleted (control) or depleted of DmRecQ4 by dsRNA from the N terminal (NRecQ4) or C terminal (CRecQ4) region DmRecQ4 were analysed by western blotting with affinity purified anti-DmRecQ4 antibody. In each case extracts from 2.5×10 5 cells were loaded. The DmRecQ4 specific band is shown in the top panel and the bottom panel shows the tubulin control. B) Equal numbers of cells were treated with dsRNA against 2 different regions DmRecQ4 (N terminal – NRecQ4 and C terminal – CRecQ4), control dsRNA (TTC4) or left untreated (no RNA) and the proliferation of the cells was measured by performing a cell count on days 3,4, 5 and 6. C) Untreated cells and Cells treated with dsRNA against 2 different regions DmRecQ4 (N terminal – NRecQ4 and C terminal – CRecQ4) or against a control dsRNA were analysed for their cell cycle distribution using FACS analysis on days 3, 4, 5 and 6. D) Untreated cells and cells which had been exposed to dsRNA corresponding the N terminus of DmRecQ4 or control dsRNA were analysed for BrdU incorporation by dot blot titration 3 days after addition of dsRNA as described in the materials and methods.

S2 cells were treated with dsRNAi against either the N or the C terminus of DmRecQ4, and the levels of the protein that remained analysed using an antibody raised against the overexpressed N terminal region of DmRecQ4. Fig1A shows that the dsRNA treatment caused at least a 90% decrease in the level of DmRecQ4 by day 3. This was maintained until at least day 7.

Cells which had been treated in this way showed markedly decreased proliferation compared to cells which had been mock treated or were untreated (Fig1B).

In addition FACS analysis of the cell cycle stage of these cells showed that by day 3 a significantly increased percentage of the cells were in S phase (Fig1C). At longer time points many cells remained in the S phase but in addition an increasing percentage of the cells showed a subG1 DNA content.

Despite the fact that a large proportion of the DmRecQ4 cells appeared to be in S phase, these cells did not appear to be actively synthesising DNA, since they showed a significant decrease in BrdU incorporation (Fig 1D). These data suggest that in S2 cells DmRecQ4 plays an important role in the progression of the cells through the S phase.

Both of these dsRNAs showed the same phenotype. The same phenotype was also seen using a third dsRNA against another different region of DmRecQ4 (data not shown). This suggests that the effects observed were specific to DmRecQ4, and not caused by off target depletion of a different protein.

To further clarify the source of the subG1 DNA content, an analysis of the amount of apoptosis occurring was carried out using an Annexin v-FITC kit and flow cytometry. This showed that on day 3 approximately 20% of all cells were annexin positive, rising to about 35% on day 4. This compares with 32% for UV treatment, 53.5% for cycloheximide treatment and 1.8% for untreated cells. Surprisingly, treatment of the RecQ4 depleted cells with the apoptosis inhibitors ALLN and ZVAD (data not shown) had no effect on the production of the subG1 content DNA suggesting that the apoptosis occurred via an apoptotic pathway which did not use caspase.

### DmRecQ4 Depleted S2 Cells Show Increased Phospho-H2AvD Staining

**Figure 2:**
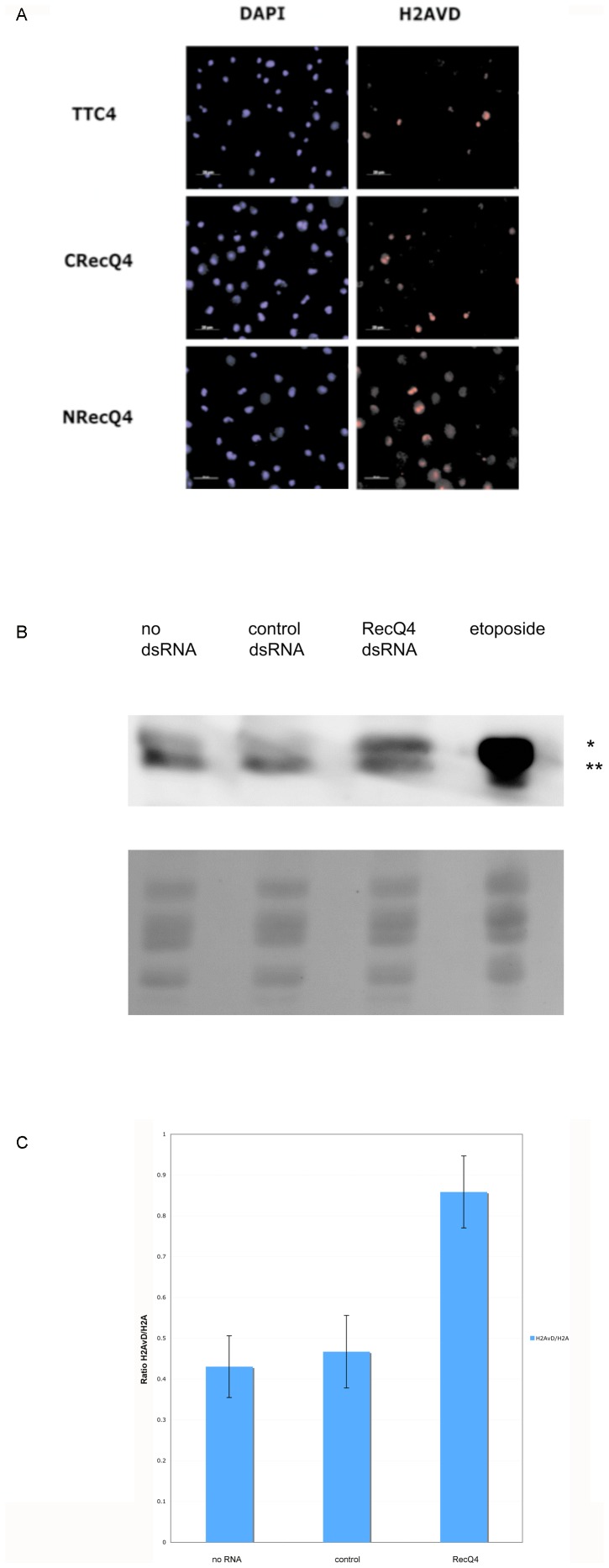
S2 cells with reduced DmRecQ4 show increases in phospho-H2AvD. A) Cells treated with dsRNA against 2 different regions DmRecQ4 (N terminal – NRecQ4 and C terminal – CRecQ4) or against a control dsRNA were fixed 4 days after dsRNA treatment and subjected to immuno-fluorescence microscopy using antibodies against Drosophila phospho-H2AvD. The DNA was visualised with DAPI. B) Chromatin was prepared from untreated S2 cells (no dsRNA) cells treated with a control dsRNA (control dsRNA) or cells treated with dsRNA against the N terminal region of DmRecQ4 (RecQ4 dsRNAi 4 days after dsRNA treatment and analysed by western blotting using an anti phospho-H2AvD antibody (top panel). This detects both phosphorylated H2AvD (*) and H2a (**). The etoposide control lane is included to allow accurate identification of the relevant bands. The bottom panel shows Ponceau red staining of the histones on the blot prior to antibody visualisation, which serves as loading control. C) Quantitation of increase in H2AvD appearance on RecQ4 depletion as detected by Western blotting. Three repetitions of the experiment were performed as described for Fig 2B. For each sample the ratio of H2AvD to H2A (which in this case serves as a loading control) was calculated using Image J.

To observe if any visible changes occurred to the chromatin on DmRecQ4 depletion, cells at day 4 were fixed and stained with DAPI to visualise the DNA, and antibodies against the phospho-H2AvD protein (the Drosophila homologue of H2Ax). Fig2A shows that more phospho-H2AvD staining was seen in DmRecQ4 depleted cells than those that had been treated with a control dsRNA. This increase could also be seen after chromatin isolation and analysis by western blotting (Fig2B/C). This suggests that DmRecQ4 depletion causes DNA damage. Similar observations have been made in S2 cells on depletion of other proteins that are known to play a role in DNA replication [19].

These observations provide further evidence for an important S phase role for DmRecQ4 in S2 cells.

### The N and C Terminal Regions are Both Important for Efficient Rescue of DmRecQ4 Depletion

**Figure 3:**
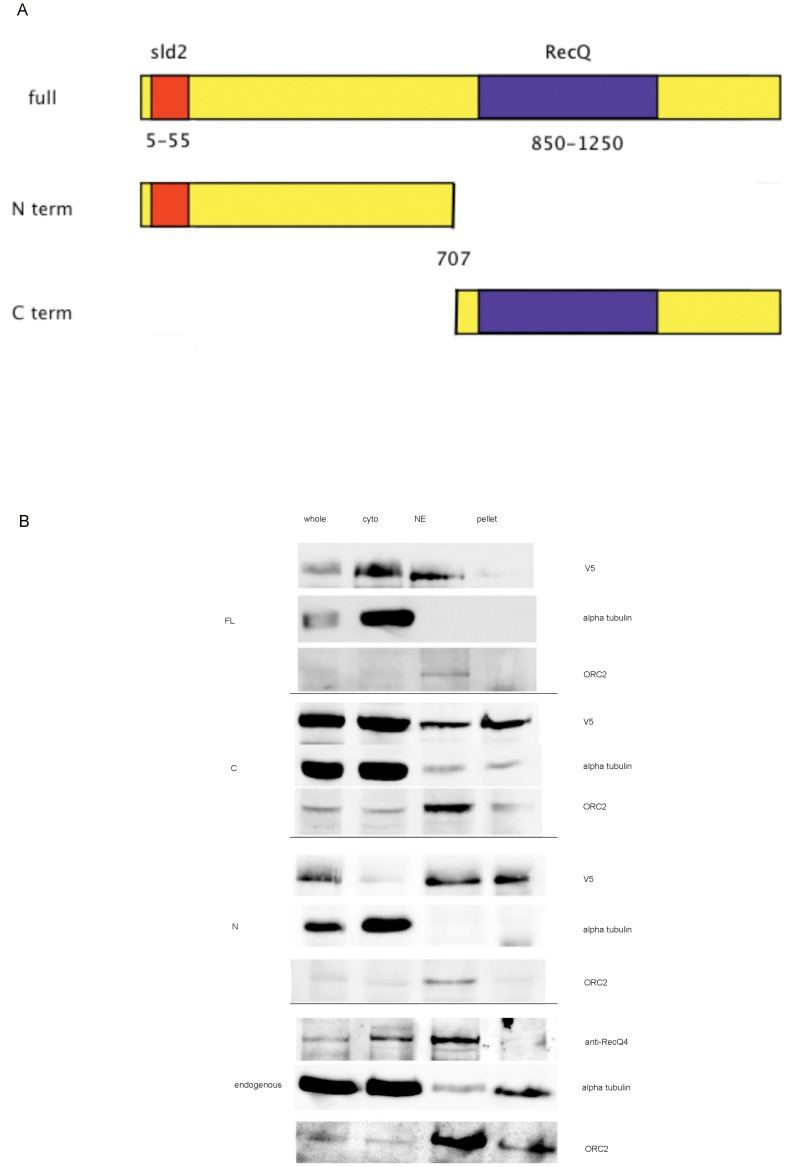
Expression of full length, N and C terminal regions in S cells. A) Diagrammatic representation of DmRecQ4 deletions constructed. B) Western blots to determine the localisation of endogenous DmRecQ4 and overexpressed full length, C terminal and N terminal fragments of DmRecQ4. Wt S2 cells or cells expressing the full length (FL), N terminal (N) or C terminal (C) DmRecQ4 were either added directly to loading buffer to make the total extract (whole) or fractionated as describe in the materials and methods to produce cytoplasmic (cyto), nuclear (NE) and pellet fractions. Western blots of these fractions were analysed with affinity purified DmRecQ4 antibody to detect the endogenous protein, V5 antibody to detect the DmRecQ4 fusion, tubulin antibody as a loading control for the whole extracts, and tubulin and Orc2 antibodies to confirm that the fractionation was successful.

The above data all support a role for DmRecQ4 in DNA replication in S2 cells, Since the N and C termini of the protein contain homology to different proteins, we were interested to determine whether both parts of the protein were required to support the replication function. It had previously been reported that in Xenopus extracts the isolated N terminus (1–596) [5], at four times higher concentration, could compensate efficiently for depletion of full length DmRecQ4 from the extract, while smaller fragments could partly compensate at much higher concentration [4]–[5]. DmRecQ4 has a 300 aa insert (228- 610) compared to Xenopus RecQ4. To cover a comparable region we therefore made Drosophila cell lines expressing full length DmRecQ4 and the isolated N (1–707) and C regions (708–1579), all of which were tagged with the V5 epitope (fig 3A).

All of these proteins were expressed in S2 cells (Fig 3B and Fig S1A), the N and C terminus at comparable levels while the full length was at a slightly lower level. Analysis of the subcellular location of these proteins by fractionation (fig3B) showed that the full length protein was distributed between the cytoplasm and nucleus. The N terminus is very tightly associated with the nucleus, while the C terminus is predominantly in the cytoplasm. This suggests that different independent elements involved in the nuclear transport of these proteins might be located in the N and C termini.

These cell lines were then assessed for their ability to withstand DmRecQ4 depletion. Initially we attempted to use a dsRNA in the upstream untranslated region of the gene to specifically deplete the endogenous protein. Unfortunately for DmRecQ4 this region is extremely short (42 bp), and therefore we were not able to get efficient DmRecQ4 depletion using only this region (data not shown). We therefore decided to look at the rescue conferred by these constructs on DmRecQ4 depletion by the two individual dsRNAs directed towards the N or C terminus of the DmRecQ4 protein. As shown (Fig S2A) the observed depletion of full length, N and C termini was as expected for these dsRNAs. No endogenous protein was visible in any of the cell lines with either the N or C terminal dsRNA. However it was still possible that small and undetectable levels remained which might influence the results. We reasoned that since the expression of the fusion proteins was comparable between the different cell lines, any titration of the added dsRNA should also be comparable. Therefore any difference in cell survival should accurately reflect the ability of the fusion constructs to compensate for the loss of the endogenous DmRecQ4 protein. Further evidence that supports this was obtained by experiments with additional mutants (see below). In addition, in one of our experiments, (see Fig S2A) the C terminal dsRNA did not fully deplete the C terminal fragment, this would therefore also be expected to be less efficient at depleting the endogenous protein. However the results of this experiment were not significantly different from those where complete depletions were observed.

**Figure 4:**
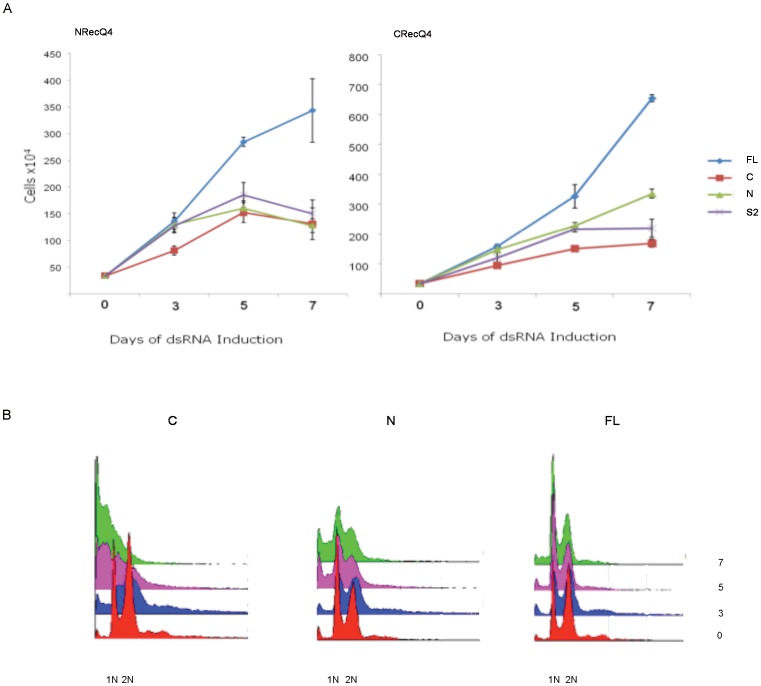
Regions in the N and C termini of DmRecQ4 are both required for complete rescue of dsRNA reduction of the endogenous protein. A) Control cells (S2) and cells expressing full length (FL), C terminal (C) and N terminal (N) fragments of DmRecQ4 were challenged with dsRNA corresponding to the N (NRecQ4) and C (CRecQ4) terminal regions and the proliferation of the cells measured by performing a cell count on days 3, 5 and 7. B) Cells expressing full length, C terminal (C) and N terminal (N) fragments of DmRecQ4 as shown were challenged with dsRNA corresponding to the C terminal regions of DmRecQ4and the cell cycle profile of the cells measured by performing FACS analysis on days 0,3, 5 and 7.

Cell growth curves for wtS2, and FL, N and C expressing cell lines that have been challenged with dsRNA against the DmRecQ4 N and C termini are shown in fig 4A. These show that while the expression of full length DmRecQ4 is able to compensate for endogenous DmRecQ4 depletion caused by either the N or the C termini, neither the N or C terminus alone is able to fully compensate for the growth defect. Minor improvements in growth can be seen with the isolated N terminus when the cells are challenged with DmRecQ4 dsDNA from the C terminal region, suggesting a possible small degree of compensation.

This is more strongly suggested by the analysis of the cell cycle behaviour of these cells by FACS (fig 4B). Here on depletion of the endogenous DmRecQ4 with dsRNA located in the C terminus the full length construct appears to revert DmRecQ4 dsRNA challenged S2 cells to a near normal cell cycle pattern, the isolated C terminus showed no rescue, while the isolated N terminus gives some small improvement in the cell cycle. The cells lines expressing the full length and isolated C terminus showed the same result using N terminal dsRNA. In this case however no rescue was seen with the cell line expressing the isolated N terminus, most likely because the levels of expressed N terminus remaining after depletion were too low to be effective.

This suggests that both the N and C termini are important for the cellular function of DmRecQ4.

### The SLD2 Domain is Required to Rescue the Replication Effects of DmRecQ4 Depletion but the Drosophila Specific Domain is not

**Figure 5:**
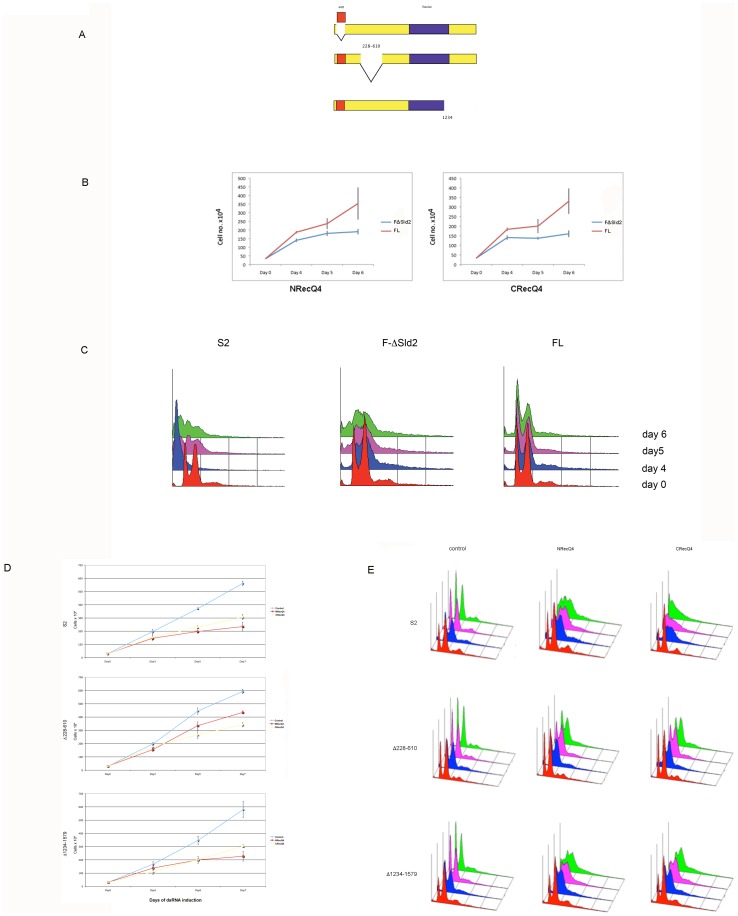
DmRecQ4 proteins without the SLD2 domain cannot rescue either cell cycle or proliferation defects while those without the Drosophila specific domain, or containing only the first 1234aa show differential effects on these parameters. A) Diagrammatic representation to show the deletions of the SLD2 homology domain, the Drosophila specific domain (228–610) and the 1234–1579 region. B) Cells expressing full length DmRecQ4 either with (red) or without (blue) the SLD2 domain were challenged with dsRNA corresponding to the N (NRecQ4) and C (CRecQ4) terminal regions of DmRecQ4. Cell proliferation was measured by cell count on days 4, 5 and 6. C) Control cells (S2) and cells expressing full length DmRecQ4 either with (FL) or without (FΔSld2) the SLD2 domain were challenged with dsRNA corresponding to the N terminal region of DmRecQ4.The cell cycle profile of the cells was measured by FACS analysis cell on days 0, 4, 5 and 6. D) Control cells (S2), cells expressing the Drosophila specific deletion DmRecQ4 (Δ228–610) and cells expressing the first 1234aa only (Δ1234–1579) were challenged with dsRNA corresponding to the N (NRecQ4) and C (CRecQ4) terminal regions of DmRecQ4. Cell proliferation was measured by cell count on days 3, 5 and 7. E) Control cells (S2), cells expressing the Drosophila specific deletion DmRecQ4 (Δ228–610) and cells expressing the first 1234aa only (D1234–1579) were challenged with dsRNA corresponding to the N (NRecQ4) and C (CRecQ4) terminal regions of DmRecQ4. The cell cycle profile of the cells was measured by FACS analysis on days 0, 3, 5 and 7.

DmRecQ4 contains a region of homology to SLD2, it might therefore be expected that this region is of importance for its replication function. The protein also contains a region from aa 228–610 which is conserved in Drosophila species but not in other organisms. Since Drosophila species lack some of the RecQ helicases, it is possible that this region plays a role in allowing Dm RecQ4 to compensate for their loss. We therefore made V5-tagged constructs of DmRecQ4 missing the SLD2 and the Drosophila specific domain (fig5A). We also made a construct covering the region 1–1234 which had been previously reported in Drosophila embryos to be able to rescue the replication defects as measured by BrdU incorporation – although only rescue viability by 10% [17]. All of these constructs were well expressed in Drosophila S2 cells (Fig S1A/B), and showed a similar subcellular distribution to that shown by the full length protein (not shown). These proteins also showed depletion patterns by the N and C terminal dsRNAs as would be expected (Fig S2B/C).

Fig 5B shows that full length protein missing the SLD2 domain has lost most of its ability to rescue the proliferation of cells treated with dsRNA against either the N or C terminus of DmRecQ4, suggesting that the SLD2 domain is important for its cellular function. Fig 5C shows that DmRecQ4 missing the SLD2 domain is also unable to significantly rescue the cell cycle profile of cells treated with dsRNA against the N terminus.

Fig 5D/E shows the comparable data for the removal of the other domains. The removal of the Drosophila specific domain Δ228–610 produced a protein that could significantly (although not completely) rescue the cell cycle phenotype generated by dsRNA treatment. Surprisingly it has a much smaller compensatory effects on viability. Further removal of the SLD2 domain from this protein removed it's ability to compensate for either. The removal of all amino acids after 1234 produced a protein which showed a slight rescue of the cell cycle phenotype while not compensating at all for the proliferation defect. Unexpectedly the removal of the Drosophila specific domain from the 1234 construct produced a protein that could not compensate for either the cell cycle or proliferation defects (not shown).

### DmRecQ4 Depleted Cells Show Abnormal Loading of Key Replication Proteins

**Figure 6:**
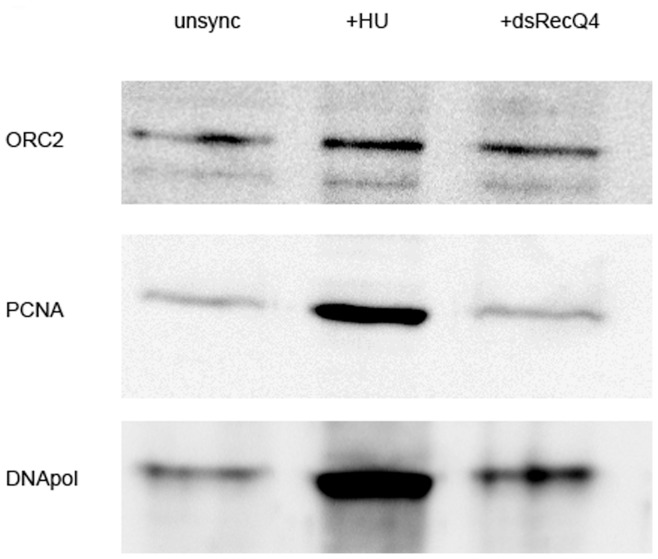
DmRecQ4 depleted cells show abnormal loading of key replication proteins. Chromatin was prepared from untreated S2 cells, cells that had been treated with HU for 24 h and cells which had been challenged with dsRNA corresponding to the N terminus of DmRecQ4 3 days after the challenge. Chromatin samples were analysed for the presence of PCNA and DNA polymerase alpha using the corresponding antibodies on western blots. In each case equal numbers of cells were subject to chromatin extraction and in addition the loading was confirmed using antibodies against the Orc2 protein.

In vitro, in Xenopus extracts, the loss of RecQ4 causes a decrease in the loading of RPA and DNA polymerase alpha onto chromatin. To determine whether this was true in vivo, we compared chromatin loaded proteins in DmRecQ4 depleted S2 cells with those in cells that had been treated with low levels of HU. The HU treatment ensures that the cells are largely in the S phase of the cell cycle. These cells also show DNA damage as measured by H2AvD labelling, and therefore should serve as a good control to see which proteins are specifically affected by DmRecQ4 loss. As expected both PCNA and DNA polymerase alpha showed a high level of loading in HU treated cells (fig 6). In contrast DmRecQ4 depleted cells on day 3 showed a marked decrease in both of these proteins, suggesting that although an S phase block was apparent from FACS analysis this was not comparable to an HU induced block. Using the same samples Orc2 shows little change in any of the samples. This suggests that the binding of Orc2 is not affected by the loss of DmRecQ4. These decreases were not caused by a decrease in the total amount of RPA or DNA polymerase in the cell (Fig S3) but are specific to chromatin bound proteins.

We also looked to see whether the reported interaction with the Mcm protein complex reported in mammalian cells could be seen in Drosophila. Immuno-precipitation of V5-DmRecQ4 from cell lines expressing the full length protein, and analysis of co-immunoprecipitating proteins using Mcm5 antibodies failed to show an interaction between these proteins (data not shown).

### Repair Functions of DmRecQ4 in S2 Cells

Although various sources have suggested a role for RecQ4 in 3 types of DNA repair (BER, HR and NER), very little is known about the molecular mechanisms of its involvement in these processes. We therefore set out to determine whether Drosophila S2 cells could provide a convenient system in which to assess this.

### DmRecQ4 Accumulates in the Nucleus soon after UV Treatment

The agent selected for investigation of the NER pathway was exposure of the cells to UV light. We first titrated the UV light to determine the level that would be applicable. Fig S4 shows the dose response of S2 cells. We subsequently chose to use a range of UV levels between 80 and 320 J/m2, as others have reported that different effects might be seen under different light intensities.

The most straightforward approach to determine whether DmRecQ4 has a role in the NER pathway would be to analyse whether DmRecQ4 depleted cells were more sensitive to UV light than control S2 cells. Unfortunately the DmRecQ4 depleted cells in the absence of UV were already so compromised that it was not possible to detect any meaningful changes on treatment of these cells with UV.

We then looked to see whether the overexpression of DmRecQ4 in addition to the endogenous protein helped the cells to resist UV treatment. Again no significant advantage in terms of cell growth and viability or recovery of cell cycle profile was observed in cells which were overexpressing the full length DmRecQ4 compared to wt S2 cells.

We also repeated the experiment with the cell lines overexpressing the isolated N and C termini of DmRecQ4. Again there was no advantage in withstanding UV damage to cells expressing either of these. In fact with the isolated N terminus at higher UV concentrations we consistently saw decreased viability of the cells with large numbers of cells appearing in the subG1 region of the FACs profile after 24 hrs (not shown).

**Figure 7:**
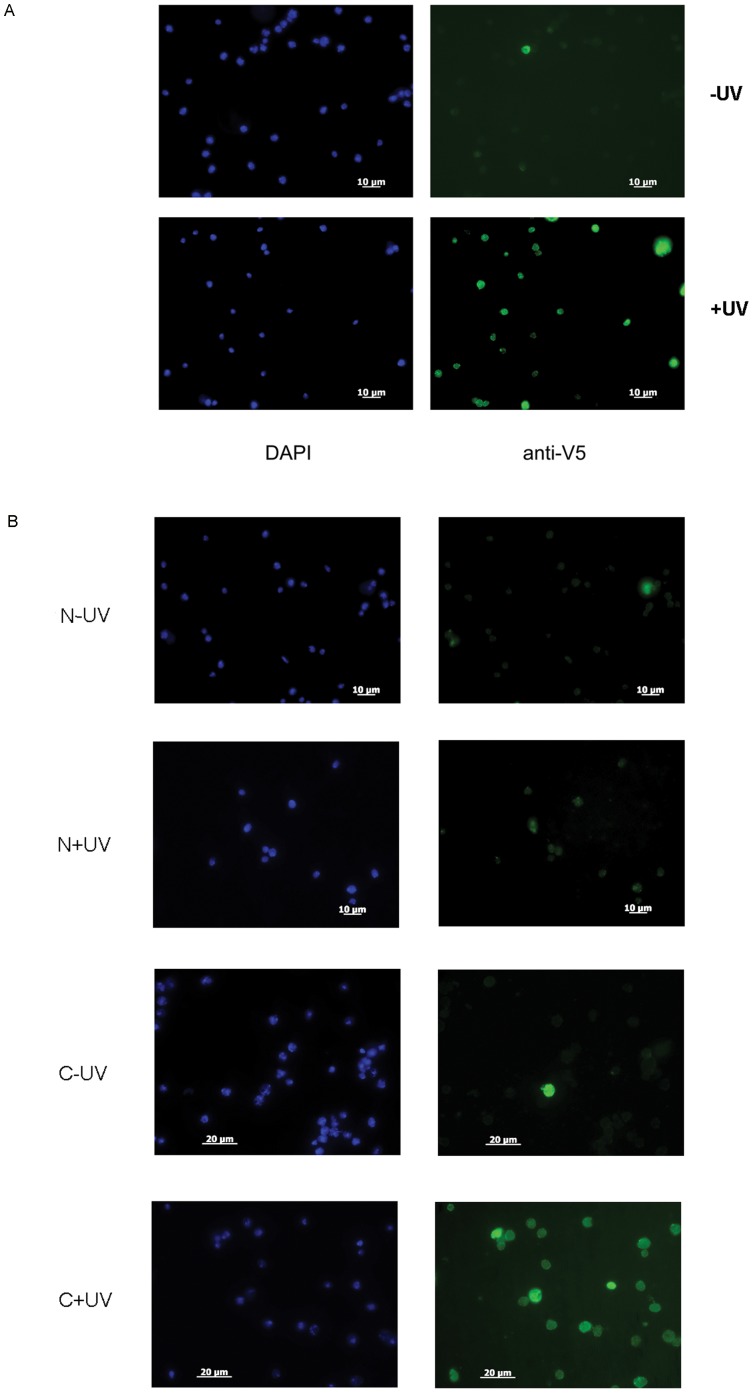
DmRecQ4 accumulates in the nucleus soon after UV treatment in a manner dependent on the presence of the C terminus. A) Untreated (top) or UV treated (80 KJ/M^2^)(bottom) S2 cells stably expressing the full length DmRecQ4 protein were subjected to detergent washes and then fixed and analysed for DNA (LHS) using DAPI or DmRecQ4 (RHS) by detection of the V5 tag on the fusion protein. B) Untreated (-) or UV treated (80 KJ/M^2^) (+) S2 cells stably expressing the isolated N (N) or C (C)) terminus of the DmRecQ4 protein were subjected to detergent washes and then fixed and analysed for DNA (LHS) using DAPI or DmRecQ4 (RHS) by detection of the V5 tag on the fusion protein.

Finally we looked to see whether we could detect a change in the DmRecQ4 subcellular distribution in response to the treatment of the cells with UV. We chose to look at the V5 tagged protein as this gave good specific staining on immunofluorescence compared to the antibody raised against the endogenous protein. The observation that it could efficiently substitute for endogenous DmRecQ4 on dsRNA interference suggested that the protein was functional. Analysis of cells fixed directly one hour after UV treatment showed no apparent redistribution of the protein on treatment with UV light at any light intensity. However as the unperturbed distribution of the protein was both nuclear and cytoplasmic we reasoned that this might obscure the visualisation of protein movement. For proteins such as the Mcm proteins it is possible to observe specific behaviour of the proteins after the cells have been extracted with detergent. We therefore subjected the UV treated cells to extraction with a detergent based solution prior to fixation and staining. As can be seen (fig7A) extraction of untreated cells with detergent caused a complete loss of DmRecQ4 staining. However in cells that had been UV treated prior to detergent extraction a strongly bound nuclear fraction could now be detected suggesting that the DmRecQ4 was being selectively retained in the nucleus. When cells were stained 5 h after UV treatment nuclear localisation of DmRecQ4 was no longer observed (not shown).

We also used the same methodology to analyse the N and C terminal fragments of DmRecQ4 (Fig 7B). This analysis showed that while the C terminus was also selectively retained in the nucleus after UV treatment, no retention of the N terminus could be detected. It should be noted that the C terminus can also be seen to be detained in the nucleus without detergent extraction (data not shown), most likely due to the fact that it is usually cytoplasmically located.

This rapid and transient re-localisation of DmRecQ4 on UV treatment suggests that it has a role in the cellular response to UV damage. The fact that this can be seen with the isolated C but not N terminus further suggests that the determinants responsible for this relocation are located in the C terminus of the protein.

### DmRecQ4 Localisation does not Change after H_2_O_2_ and Etoposide Treatment

**Figure 8:**
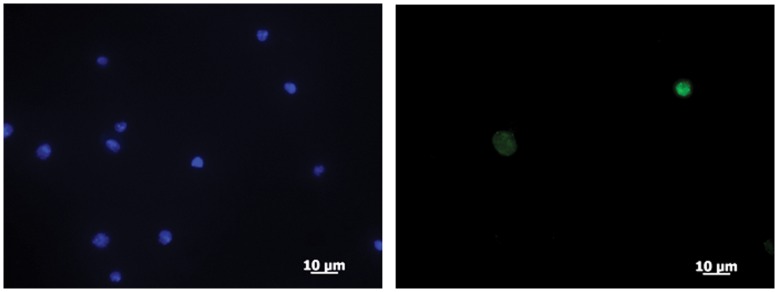
DmRecQ4 localisation does not change after H_2_O_2_ or etoposide treatment. S2 cells expressing the full length DmRecQ4 protein 1 h after exposure to etoposide were subjected to detergent washes and then fixed and analysed for DNA (LHS) using DAPI or DmRecQ4 (RHS) by detection of the V5 tag on the fusion protein.

Etoposide is often used as an agent to study HR since it generates replication dependent ds DNA breaks. For BER a commonly used agent is H_2_O_2_. Dose response curves for both of these reagents are shown in figures S5/S6. From these data we decided to use etoposide at 1 uM which left some percentage of the cells capable of proliferation and 10 uM to provide a more lethal dosage. We chose to use H_2_O_2_ at 20 mM. Since UV damage caused nuclear retention of RecQ4, it was possible that the same would be true for other forms of damage we therefore chose to use this as our primary method of analysis for etoposide and H_2_O_2_ treatment. Immuno-fluorescence analysis of detergent extracted cells expressing the FL and the N, and C termini one hour or five hours after etoposide or H_2_O_2_ treatment did not show nuclear retention of any of these proteins. Fig 8 shows this data for the full length DmRecQ4 treated with etoposide after one hour, but similar results were obtained with the full length protein after H_2_O_2_ treatment and the isolated N and C termini after either treatment (not shown) at both time points.

One of the main pieces of evidence for the involvement of RecQ4 in each of these pathways is its interaction with proteins specific to that pathway – XPA for NER, Rad51 for HR, FEN and APE1 for BER. We therefore reasoned that different pathways might be involved in its localisation for each of these pathways. It was therefore possible that nuclear retention in each case might be differentially sensitive to detergent treatment. We therefore repeated the immuno-fluorescence in the absence of prior detergent extraction with the C terminal fragment. For this construct nuclear retention can be seen after UV treatment when the cells are untreated prior to fixation, however neither etoposide or H_2_O_2_ produced a similar localisation.

## Discussion

### DmRecQ4 in DNA Replication

Our results show that in Drosophila S2 cells, the loss of the DmRecQ4 protein produces phenotypes that are consistent with a role in DNA replication. The cells show reduced proliferation, an S phase block, and a decrease in BrdU incorporation. That this is a direct effect on replication, and not a secondary effect, is suggested by the observed decreases in the loading of the DNA polymerase alpha and PCNA. These results are in agreement with previous observations in Xenopus in vitro extracts [4]–[5]. The decrease in replication protein loading further suggests that the reduced levels of BrdU incorporation are due to a decrease in the number of active forks, rather than a reduced rate of fork movement. This could be due to decreased origin firing or an increased instability of forks, however our data does not allow us to distinguish between these possibilities, as both have been shown to cause an increase in the appearance of chromatin bound H2Ax (H2AvD). In mammalian cells it has been suggested that the main role of RecQ4 is in replication restart after disruption of the forks [6]. However the relevance of this suggestion to Drosophila is unclear since studies in whole flies suggest that DmRecQ4 mutants show no defect on HU treatment [17].

Some have reported an interaction of RecQ4 with the Mcm2-7 complex [6]–[7] while others are not able to detect this interaction [4]–[5]. In S2 cells we do not see any interaction of DmRecQ4 with the Mcm proteins by immuno-precipitation. This suggests either that there is no interaction or that the stability of the complex varies depending on the organism under consideration.

Our results are consistent with previous studies in Drosophila larvae [17] showing that depletion of DmRecQ4 caused decreased proliferation and BrdU incorporation. In addition, our results extend these studies by showing that the effects on BrdU incorporation are mediated directly via loss of the chromatin binding of key replication proteins.

We also show that depletion of DmRecQ4 in S2 cells produces a significant percentage of cells which have subG1 DNA content. This is consistent with a role for RecQ4 in replication, as the loss of several other replication proteins has been reported to generate subG1 DNA content via apoptosis or premature mitosis e.g. It is possible that some of the subG1 content is caused by a failure to activate a checkpoint arrest. However the annexin assays suggest that most of the DNA fragmentation is likely to be due to apoptosis, although the surprising observation that the subG1 levels observed were refractory to inhibition by a broad range caspase inhibitor ZVAD and the calpain inhibitor ALLN suggests that the apoptosis may be via a non standard pathway. Apoptosis has also been reported for the loss of RecQ4 in DT40 cells [20].

### Regions of DmRecQ4 Required for Replication

Loss of the SLD2 domain, either by deletion of the whole N terminus or specific removal of the SLD2 domain, suggests that the replication function of DmRecQ4 requires the presence of the SLD2 domain. However this region is not sufficient for the replication function, as depletion of endogenous DmRecQ4 protein is only partly rescued by a mutant protein containing residues 1–1234, or to a slightly lesser extent 1–707. This suggests that regions in the C terminus of the protein are also needed for progression through S phase. This data is consistent with previous work in other systems. In Xenopus in vitro extracts, addition of an excess of N terminal fragments gave a partial recovery of activity (aa1-118 at 100 fold excess [4] or aa1- 596 at 4 fold excess) [5]. In fly embryos expression of DmRecQ4 aa1-1234 could rescue loss of BrdU incorporation although only rescued the viability by 10% [17]. In contrast, in chicken DT40 cells [20] and the human pre-B cell line Nalm-6 [21] the N terminus alone can support DNA replication in the absence of DNA damage.

The use of S2 cells has also allowed us to demonstrate that various deletion mutants show differential rescue of proliferation and replication phenotypes. Although our data does not completely rule out the possibility that some of the effects observed are due to misfolding of the protein, this suggests that DmRecQ4 has more than one important cellular function, and that these constructs are only able to compensate for a subset of the functions. Two of the deletions showing this effect interfere with the RecQ helicase domain, suggesting that this might be a repair associated function. The other mutation which shows this phenotype removes a 300aa Drosophila specific section of the protein. This could also be consistent with possible additional repair roles for the DmRecQ4, due to the decreased number of RecQ proteins in that organism.

### DmRecQ4 in DNA Repair

The rapid retention of DmRecQ4 in the nucleus after UV treatment provides good evidence for a role for the protein in the response to UV damage in S2 cells. The observation that a similar movement does not occur after etoposide and H_2_O_2_ treatment suggests that this is likely to be specific for NER, and not just due to the fact that the cells are arrested by a damage checkpoint. The ability of the isolated C terminus to carry out the same relocation further suggests that determinants for this are located in the C terminus of the protein.

The lack of nuclear retention in response to etoposide and H_2_O_2_ is somewhat surprising since previous studies in vertebrates have proposed that RecQ4 is involved in NER, BER and HR. Experiments in Xenopus egg extracts suggested that RecQ4 was involved in the repair of dsbs [22]. In addition Human nalm-6 cells expressing a version of RecQ4 lacking the C terminus were hypersensitive to ionizing radiation [21].Furthermore, in mammalian cells, RecQ4 relocalisation to the nucleus was seen after H_2_O_2_ treatment [12] [23], but not after treatment with bleomycin, etoposide, UV irradiation and gamma irradiation [23]. Previous studies in Drosophila larval brains did not address NER, however they did show that DmRecQ4 mutants were more sensitive to paraquat (which generates oxidative damage repaired by BER), a single dsb, and gamma irradiation (which mainly induces dsb repair but also generates oxidative damage and ss DNA breaks). One possible reason for these differences might be that the involvement of RecQ4 in these damage response pathways is less direct. In flies the assay used was viability, which was measured several days after the mutagen was applied. If RecQ4 deficiency caused a defect downstream from the actual repair process, viability would be affected but no RecQ4 movement would be seen directly after damage. Such a defect could be the premature mitosis events which we observe in S2 cells, and which is also consistent with the severely compromised genomic integrity observed in larval brains [17]. Alternatively since each of these pathways involves a largely unique set of proteins, it is possible that the involvement of RecQ4 in each pathway involves an interaction with a different protein. These interactions may have very different characteristics, for instance they may be more or less transient/stable, and may involve different regions of the protein. Our observation therefore does not rule out a role for RecQ4 in other pathways, but does suggest that the mechanism of transport to or retention in the nucleus is different in each case.

## Materials and Methods

### Antibodies and Reagents

V5 and PCNA mouse monoclonal antibodies were from Abcam. Rabbit antiBrdU and mouse anti-tubulin monoclonal antibodies were from Sigma. Rabbit anti phospho-H2AvD was obtained from Rockland and Mouse anti-lamin was a gift from D. Glover. Rabbit antibodies against DNA polymerase alpha were as previously reported [24]. Antibodies against RecQ4 were manufactured in guinea pigs using a his-tagged 371 amino acid region from the N terminus of the protein and before use were affinity purified against the overexpressed RecQ4 protein.

HRP labelled secondary antibodies used for western blotting were obtained from Thermo Scientific (anti-rabbit and anti-mouse) and Jackson (anti-guinea pig). Secondary antibodies for immunostaining (Alexa 594 anti-rabbit and Alexa 488 anti-mouse) were from Molecular Probes, Oregon, USA.

ALLN was from Enzo life sciences, ZVAD from Calbiochem and Etoposide and HU from Sigma.

### Drosophila S2 Cells

S2 cells (originally obtained from the Drosophila Genomics Resource Center) were grown in Schneiders Drosophila medium from Lonza, with 10% Foetal calf serum from Gibco and penicillin/streptomycin from Sigma.

### DsRNA Interference

These were performed as described previously [25]. Briefly two non-overlapping regions of DmRecQ4 mRNA were chosen as the targets for the RNA interference experiment. The N terminal region was amplified using the primers GCAAAGCCCAGGAGTACAAG at the 5′ end and TTGCGCTTTGCCTTATCTTT at the 3′ end. The C terminal region (was amplified using the primers GAAGCTGGAGAACGCATAG at the 5′ end and G CAACAAGCTGTCTCCCTTC at the 3′ end. These primers were all made to contain a 5′ T7 RNA polymerase binding site. The T7 sites were then utilised to make dsRNA using the MEGAscript T7 kit (Ambion) as per manufacturers instructions. The dsRNA interference experiment was carried out on S2 cells in exponential growth phase using 10 µg of dsRNA per 10^6^ cells and the cells were monitored by cell count, FACS analysis and protein blotting over a period of 7 days.

### Overexpression of DmRecQ4 and Deletions in S2 Cells

A full length cDNA clone for DmRecQ4 was constructed from its corresponding genomic region by the removal of introns using pcr. This was cloned into the Xho1 and Nco1 sites of the pMT/V5 hisA vector (Invitrogen) in such a way that it was His- and SV5-tagged at the C terminus and under the control of the inducible metallothionein promoter. The construct was introduced into S2 cells along with the pCoBlast vector using the calcium phosphate procedure, and cells stably transfected with the DmRecQ4 gene were selected using blasticidin according to the manufacturer's instructions.

Deletion constructs were made by removing the region required using internal pcr and intramolecular ligation.

### Measurement of BrdU Incorporation by Dot Blot

This was carried out largely as previously described [26]. S2 cells at day 3 post RNAi treatment were labelled with BrdU (20 µM) for 1 h. The cells were harvested, resuspended in RSB buffer (10 mM Tris-HCl pH8, 10 mM NaCl, 3 mM MgCl2) at a concentration of 2.5×10^7^ cells/ml, and incubated on ice for 5 min. An equal volume of 0.2% NP-40 in RSB buffer was added followed by incubation in ice for an additional 10 min. The nuclei obtained were pelleted by centrifugation (5000 xg for 5 min) and resuspended in 3 ml lysis buffer (200 mM NaCl, 10 mM Tris-HCl pH8, 25 mM EDTA, 1% SDS and 100 µg/ml proteinase K (Roche)) overnight at 37°C. The sample was then extracted twice with phenol:chloroform:isoamyl alcohol (25∶24:1). After extraction, an equal volume of isopropanol was added to the aqueous phase and the precipitate was collected by centrifugation for 30 min (16000 xg for 30 mins) at 4°C. The DNA was resuspended in TE buffer (10 mM Tris-HCl pH 8, 1 mM EDTA) and the concentration was measured by spectrophotometry.

To denature the sample 5 µl of the DNA solution (at 1 µg/µl) was mixed with 45 µl of NaOH 0.4N, vortexed and incubated for 30 minutes on ice. The solution was neutralised by addition of 50 µl of 1M Tris-HCl pH8. Then a dilution series of the final solution was spotted on nitrocellulose: Amersham Hybond ECL (G.E). The negative control was DNA extracted from S2 cells (no BrdU labelling). The nitrocellulose membrane was incubated overnight in PBS+1% Tween+1% perfect block (Mo Bi Tech) and incubated with primary (anti BrdU) and secondary antibodies as for protein blotting. Quantification was performed using the Image gauge software on a Fujifilm Life Science LAS-4000 imaging system (Fuji).

### Flow Cytometry

Cells were harvested and fixed using 50% ethanol in PBS. Immediately prior to use cells were resuspended in PBS containing 1% glucose, 10 µg/ml RNase,1 mM EDTA,0.5% Triton X100 and 50 µg/ml propidium iodide to stain DNA. Flow cytometry was carried out on a CYTOMICS 500 (Coulter Beckman) analysis was done using CXP software.

### Cell Fractionation

Cell fractionation was carried out using the Proteojet cytoplasmic and nuclear protein extraction kit (Fermentas) following the manufacturers instructions. All steps were carried out at 4°C. Briefly cells were collected by centrifugation at 6,500 rpm and washed twice with PBS. They were resuspended in cold cell lysis buffer supplemented with DTT and protease inhibitors, incubated on ice for 10 minutes and centrifuged at 13,000 rpm. The supernatant was re-centrifuged and the resulting supernatant is the cytoplasmic fraction. The nuclear extract was obtained by incubating the nuclear pellet in nuclear lysis buffer for 15 minutes followed by centrifugation. The supernatant after centrifugation is the nuclear extract and the pellet which was resuspended in SDS PAGE loading buffer constitutes the pellet fraction.

### Chromatin Extraction

All steps were carried out at 4°C. Cells were collected by centrifugation at 6,500 rpm, washed with cold PBS and re-suspended in chromatin buffer (PBS, 0.5% triton, 2.5 mM MgCl2, 10 µg/ml proteinase inhibitor). After 5 min incubation on ice the pellet was recovered by centrifuging for 5 minutes at 6,500 rpm and re-suspended in 1X SDS-PAGE loading buffer at a concentration equivalent to 250,000 cells/µl.

### Protein Blotting

Proteins from SDS PAGE were blotted onto Amersham Hybond ECL (G.E) and developed with Immobilon Western Chemiluminescent HRP Substrate(Millipore) Visualisation and quantitation were carried out using Fujifilm Life Science LAS-4000 imaging system (Fuji).

### Immunofluorescence

An aliquot of cells was deposited on polylysine treated coverslips. The cells were fixed using 4% paraformaldehyde in 1.1 mM Na_2_HPO_4_, 0.4 mM KH_2_PO_4_, 137 mM NaCl, 5 mM KCl, 2 mM MgCl_2_, 2 mM EGTA, 5 mM Pipes, 5.5 mM glucose, pH 6.1 [27] Cells were permeabilised in PBS, 1% BSA and 0.1% triton X100, the coverslips were processed for immuno-fluorescence using the appropriate antibodies described in the figure legends. The DNA was counterstained with DAPI. The coverslips were mounted in mounting medium Vectashield (Vector), and analysed.

Where Triton extraction was used this was carried out by incubating the cells in a buffer containing 0.2% triton for 5 minutes prior to fixation.

### Apoptosis Assays

Cells from days 3/4 of the dsRNA treatment were collected and analysed using the Annexin V-FITC apoptosis detection kit from Sigma (cat no. APOAF). For the positive controls S2 cells were treated with 10 uM cycloheximide for 5 h and analysed, or 90J of UV and analysed 5h after treatment.

### UV Treatment

Exponentially growing S2 cells were collected by centrifugation and resuspended in PBS to prevent quenching. They were subjected to UV irradiation using a hand held UV light. The amount of radiation received was calibrated using a UVX radiometer.

### Etoposide and H_2_O_2_ Treatment

Exponentially growing S2 cells were exposed to etoposide or H_2_O_2_ by the addition of DNA damaging agent directly to the growing medium.

## Supporting Information

Figure S1 **Expression levels of DmRecQ4 full length and deletion mutants.** A. Whole cell extracts from cells expressing full length RecQ4 (FL) the N terminal region (N), the C terminal region (C), the N terminal region from which the SLD2 domain has been removed (NΔSld2), the full length RecQ4 from which the SLD2 domain has been removed (FLΔSld2), RecQ4 with a deletion of 228–610 (Δ228–610) and with the first 1234 amino acids of the protein (Δ1234–1579) were analysed for the presence of the V5 antigen by western blot. In each case equal numbers of cells were loaded and the loading was checked by tubulin staining. B. Whole cell extracts from wild type S2 cells and S2 cell lines expressing the first 1234 amino acids of the protein (Δ1234–1579), expressing RecQ4 with a deletion of 228–610 (Δ228–610) and with this deletion plus a deletion of the SLD2 domain (Δ228 ΔSld2) were analysed for the presence of the RecQ4 protein by western blotting using the affinity purified anti-RecQ4 antibody. The relevant bands are marked (*)
(TIF)

Figure S2 **Efficiency of depletion of full length DmRecQ4 and deletion mutants with dsRNA corresponding to the N and C termini of the protein.** A. Whole cell extracts from cells expressing full length RecQ4, and the C and N terminal regions were analysed for the presence of the V5 antigen in the presence of dsRNA corresponding to the N terminus (N = NRecQ4) and C terminus (C = CRecQ4) at days 3, 5 and 7 as shown. Each set includes a negative control of untreated S2 cells and, in the case of the full length only, a positive control of cells treated with a dsRNA against a control DNA (con). The top panel shows V5 antigen and the bottom the corresponding tubulin control. In each case A and B corresponds to two independent repetitions of the experiment. B. Whole cell extracts from cells expressing full length RecQ4 (FL) and RecQ4 without the SLD2 domain (DSld2) were analysed for the presence of the V5 antigen in the presence of dsRNA corresponding to the N terminus (N) and C terminus (C) at days 4, 5 and 6 as shown. Each set includes a positive control of cells treated with a dsRNA against a control DNA (con). The top panel shows V5 antigen and the bottom the corresponding tubulin control. In each case 1 and 2 corresponds to two independent repetitions of the experiment. C. Whole cell extracts from S2 cell lines expressing the first 1234 amino acids of the protein (Δ1234–1579), RecQ4 with a deletion of 228–610 (Δ228–610), and with Δ228–610 plus a deletion of the SLD2 domain (Δ228 ΔSld2) were analysed for the presence of the V5 antigen in the presence of dsRNA corresponding to the N terminus (N) and C terminus (C) on day 5. Each set includes a positive control of cells treated with a dsRNA against a control DNA (con). The top panel shows V5 antigen and the bottom the corresponding tubulin control. 1 and 2 corresponds to two independent repetitions of the experiment.
(TIF)

Figure S3 **Reduction in the level of DmRecQ4 does not alter the overall expression of PCNA or DNA polymerase a.** Whole cell extracts from untreated S2 cells (no dsRNA), cells treated with control dsRNA (control dsRNA) and cells treated with dsRNA corresponding to the N terminus of RecQ4 (RecQ4 dsRNA) were analysed for the presence of PCNA and DNA polymerase alpha using the corresponding antibodies on western blots. Below each western is shown the corresponding ponceau stain of the membrane prior to blotting to show that equal amounts of protein have been loaded in each lane.
(TIF)

Figure S4 **Wt S2 cells were subjected to various levels of UV as shown and the percentage of the cells in subG1 phase analysed by FACS analysis after 1, 5, 23, 27 and 43h.**
(TIF)

Figure S5 **Wt S2 cells were subjected to various levels of etoposide (uM) as shown and the proliferation of the cells analysed by cell count after 1, 19, 25 and 43h.**
(TIF)

Figure S6 **Wt S2 cells were subjected to various concentrations of H_2_O_2_ (mM) as shown and the proliferation of the cells analysed by cell count after 1, 5, 23, 29 and 45h.**
(TIF)
