# Supplementary figures and images for: Drosophila RecQ4 Is Directly Involved in Both DNA Replication and the Response to UV Damage in S2 Cells
Source: PLoS One. 2012 Nov 16;7(11):e49505. doi: 10.1371/journal.pone.0049505 (PMC3500301; doi:10.1371/journal.pone.0049505)

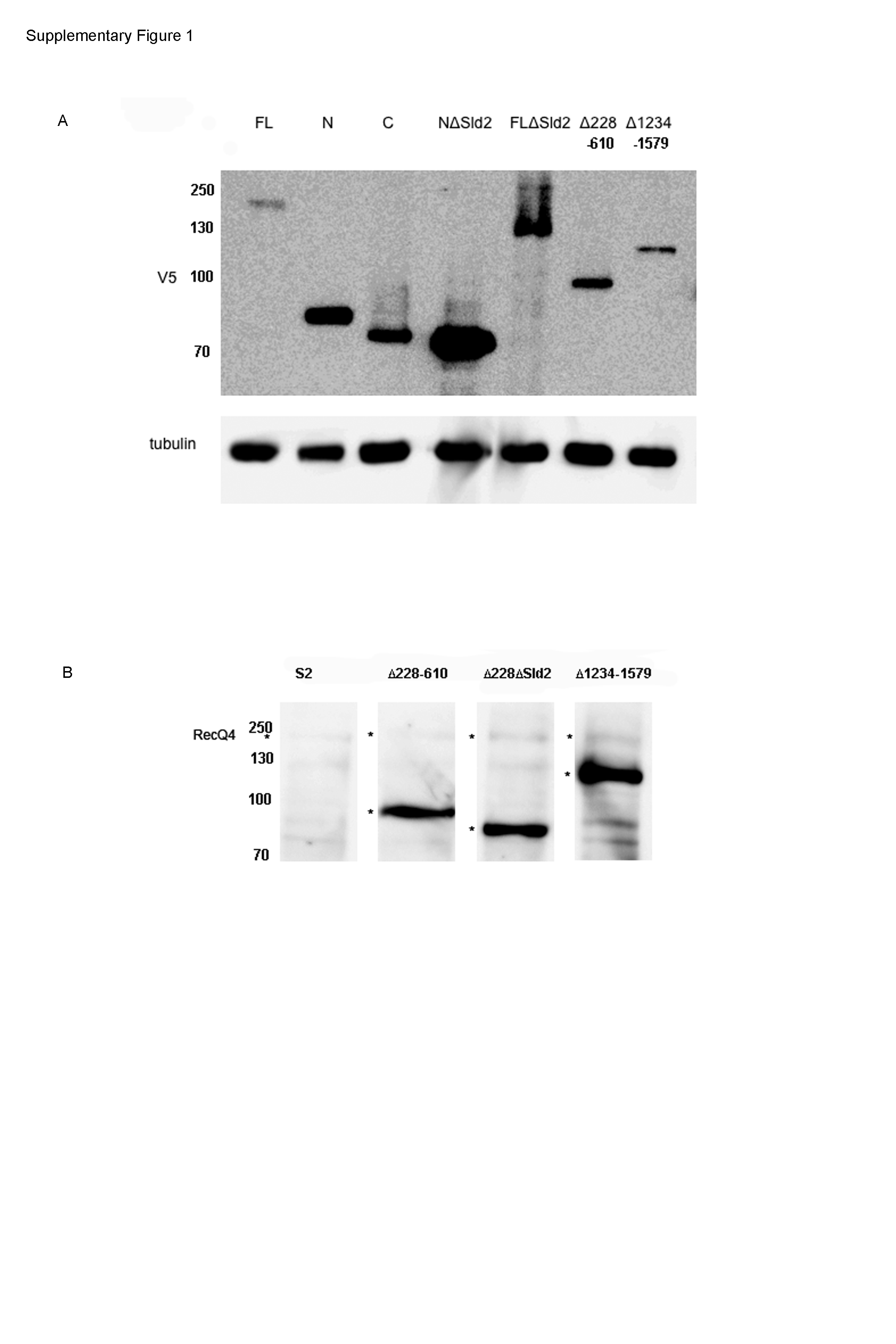

Supplement: Figure S1 — Expression levels of DmRecQ4 full length and deletion mutants. A. Whole cell extracts from cells expressing full length RecQ4 (FL) the N terminal region (N), the C terminal region (C), the N terminal region from which the SLD2 domain has been removed (NΔSld2), the full length RecQ4 from which the SLD2 domain has been removed (FLΔSld2), RecQ4 with a deletion of 228–610 (Δ228–610) and with the first 1234 amino acids of the protein (Δ1234–1579) were analysed for the presence of the V5 antigen by western blot. In each case equal numbers of cells were loaded and the loading was checked by tubulin staining. B. Whole cell extracts from wild type S2 cells and S2 cell lines expressing the first 1234 amino acids of the protein (Δ1234–1579), expressing RecQ4 with a deletion of 228–610 (Δ228–610) and with this deletion plus a deletion of the SLD2 domain (Δ228 ΔSld2) were analysed for the presence of the RecQ4 protein by western blotting using the affinity purified anti-RecQ4 antibody. The relevant bands are marked (*) (TIF) [file pone.0049505.s001.tif]

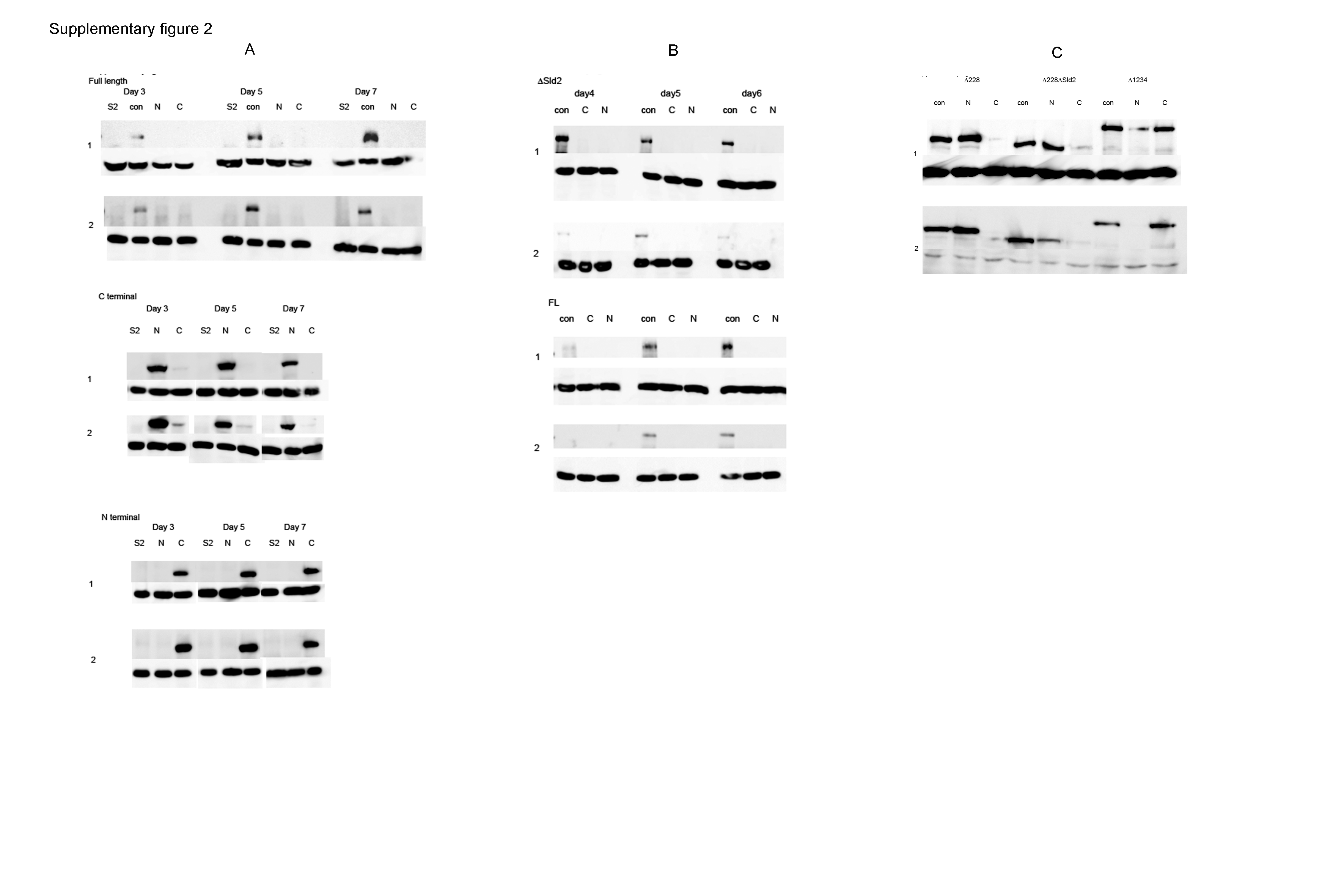

Supplement: Figure S2 — Efficiency of depletion of full length DmRecQ4 and deletion mutants with dsRNA corresponding to the N and C termini of the protein. A. Whole cell extracts from cells expressing full length RecQ4, and the C and N terminal regions were analysed for the presence of the V5 antigen in the presence of dsRNA corresponding to the N terminus (N = NRecQ4) and C terminus (C = CRecQ4) at days 3, 5 and 7 as shown. Each set includes a negative control of untreated S2 cells and, in the case of the full length only, a positive control of cells treated with a dsRNA against a control DNA (con). The top panel shows V5 antigen and the bottom the corresponding tubulin control. In each case A and B corresponds to two independent repetitions of the experiment. B. Whole cell extracts from cells expressing full length RecQ4 (FL) and RecQ4 without the SLD2 domain (DSld2) were analysed for the presence of the V5 antigen in the presence of dsRNA corresponding to the N terminus (N) and C terminus (C) at days 4, 5 and 6 as shown. Each set includes a positive control of cells treated with a dsRNA against a control DNA (con). The top panel shows V5 antigen and the bottom the corresponding tubulin control. In each case 1 and 2 corresponds to two independent repetitions of the experiment. C. Whole cell extracts from S2 cell lines expressing the first 1234 amino acids of the protein (Δ1234–1579), RecQ4 with a deletion of 228–610 (Δ228–610), and with Δ228–610 plus a deletion of the SLD2 domain (Δ228 ΔSld2) were analysed for the presence of the V5 antigen in the presence of dsRNA corresponding to the N terminus (N) and C terminus (C) on day 5. Each set includes a positive control of cells treated with a dsRNA against a control DNA (con). The top panel shows V5 antigen and the bottom the corresponding tubulin control. 1 and 2 corresponds to two independent repetitions of the experiment. (TIF) [file pone.0049505.s002.tif]

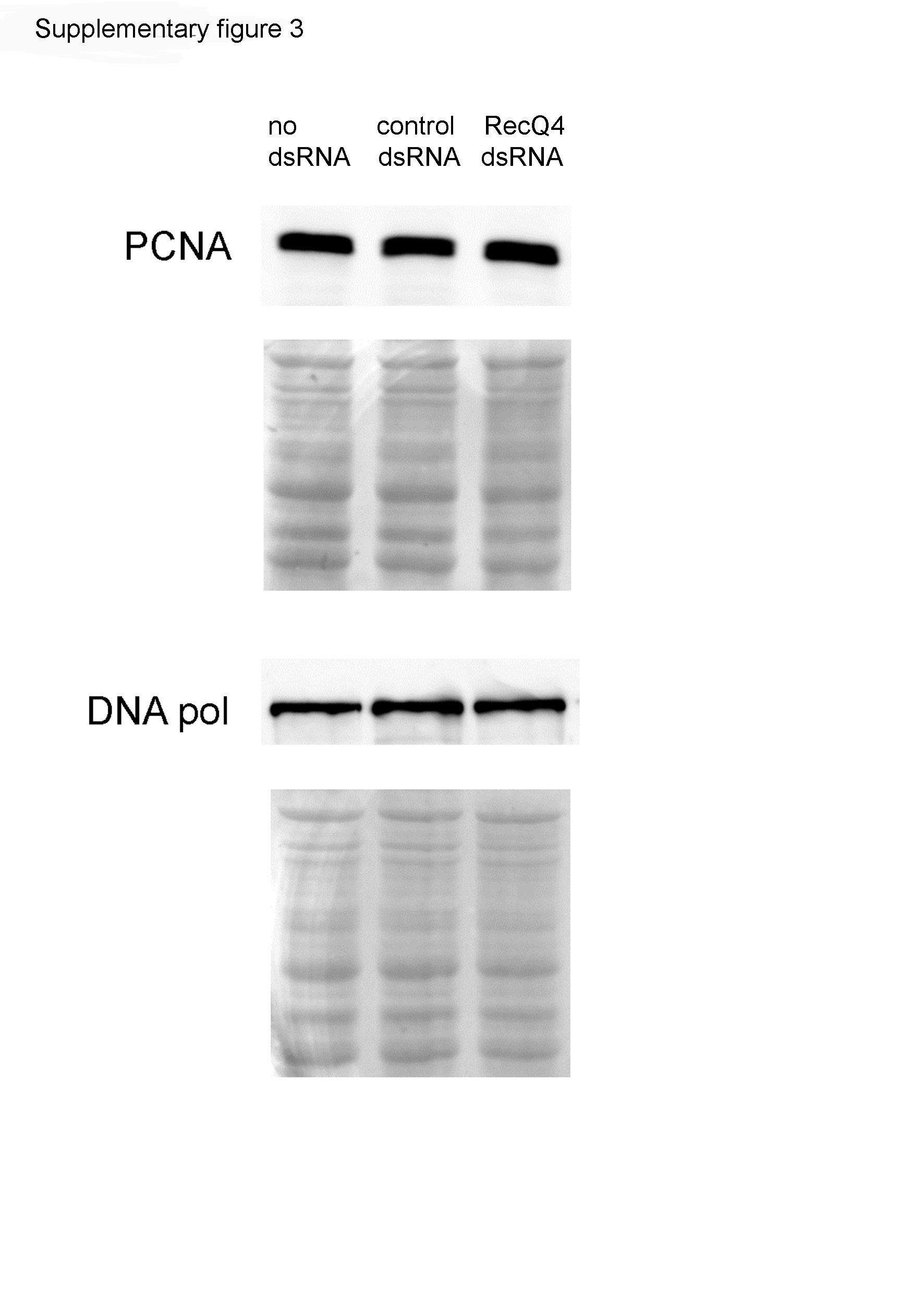

Supplement: Figure S3 — Reduction in the level of DmRecQ4 does not alter the overall expression of PCNA or DNA polymerase a. Whole cell extracts from untreated S2 cells (no dsRNA), cells treated with control dsRNA (control dsRNA) and cells treated with dsRNA corresponding to the N terminus of RecQ4 (RecQ4 dsRNA) were analysed for the presence of PCNA and DNA polymerase alpha using the corresponding antibodies on western blots. Below each western is shown the corresponding ponceau stain of the membrane prior to blotting to show that equal amounts of protein have been loaded in each lane. (TIF) [file pone.0049505.s003.tif]

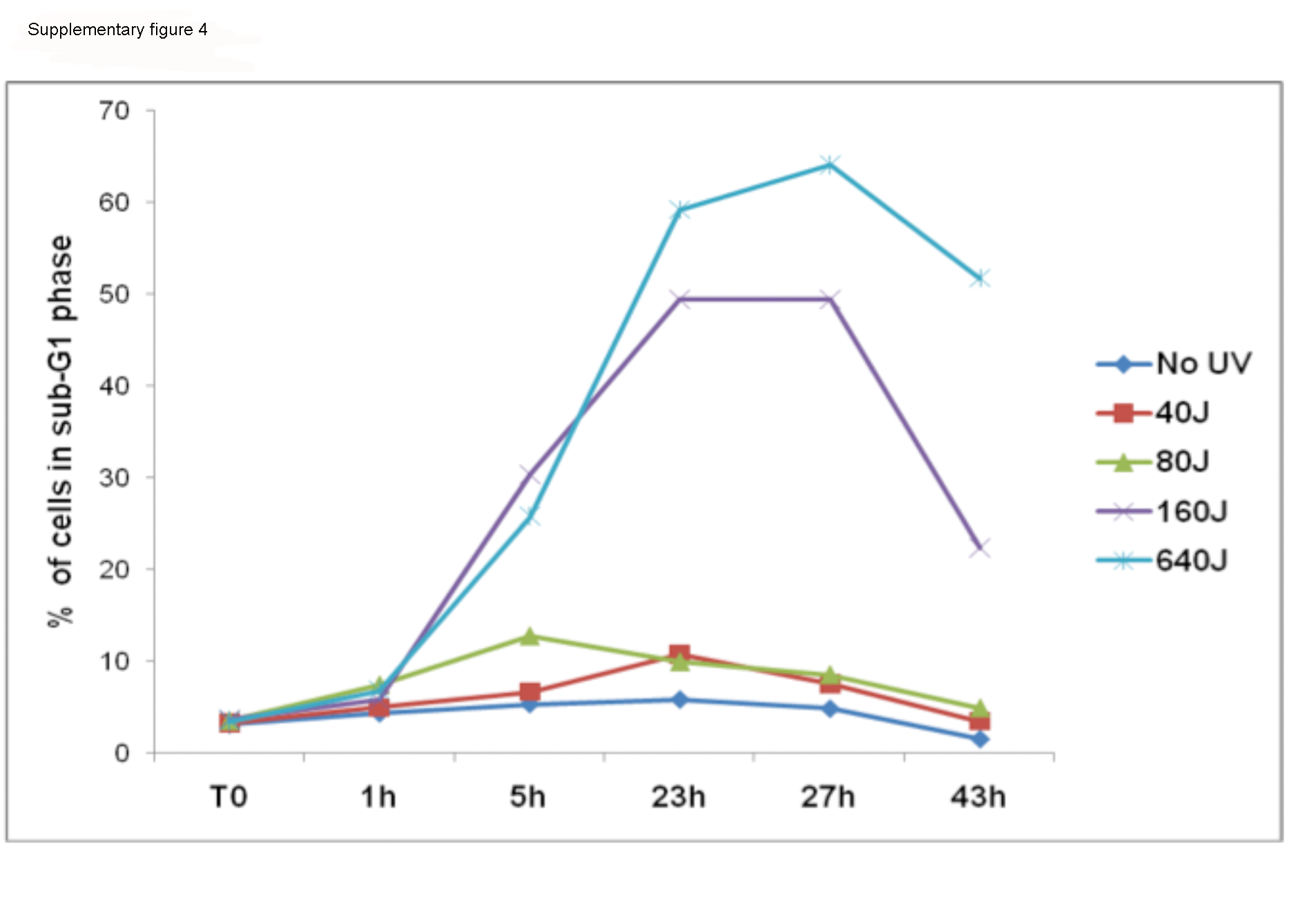

Supplement: Figure S4 — Wt S2 cells were subjected to various levels of UV as shown and the percentage of the cells in subG1 phase analysed by FACS analysis after 1, 5, 23, 27 and 43h. (TIF) [file pone.0049505.s004.tif]

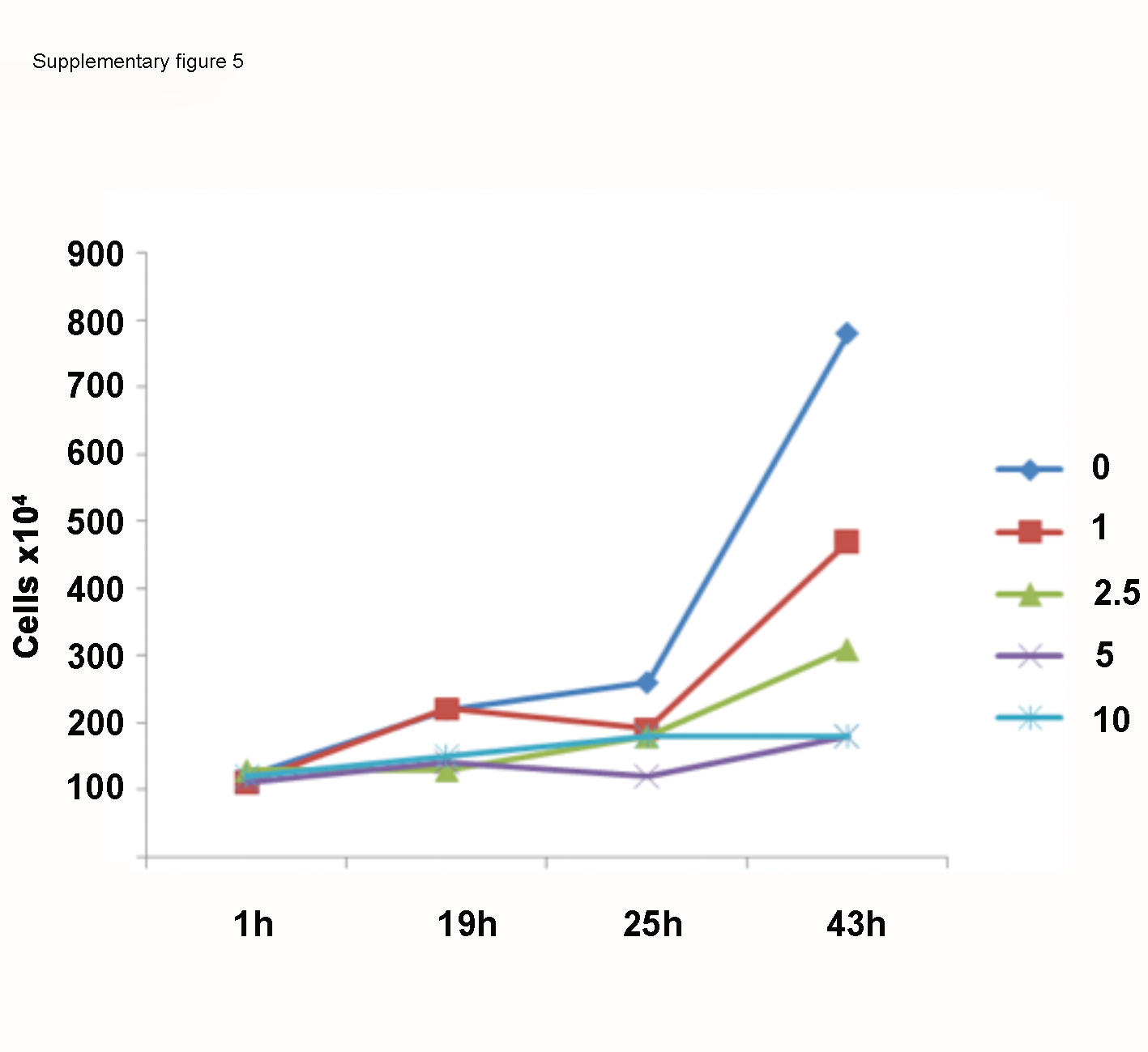

Supplement: Figure S5 — Wt S2 cells were subjected to various levels of etoposide (uM) as shown and the proliferation of the cells analysed by cell count after 1, 19, 25 and 43h. (TIF) [file pone.0049505.s005.tif]

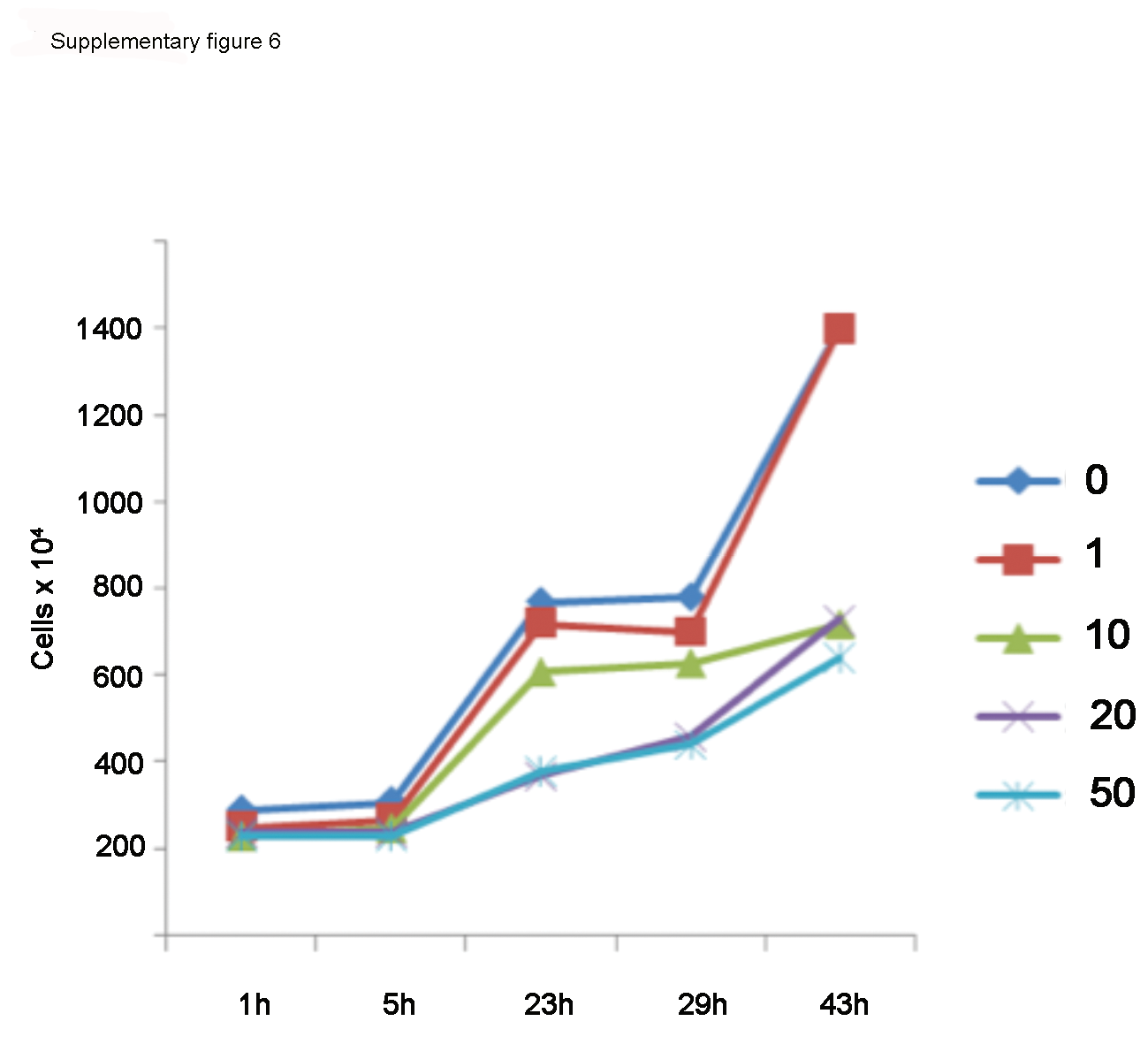

Supplement: Figure S6 — Wt S2 cells were subjected to various concentrations of H2O2 (mM) as shown and the proliferation of the cells analysed by cell count after 1, 5, 23, 29 and 45h. (TIF) [file pone.0049505.s006.tif]
